# Tumor-derived Matrix Metalloproteinase-13 (MMP-13) correlates with poor prognoses of invasive breast cancer

**DOI:** 10.1186/1471-2407-8-83

**Published:** 2008-03-28

**Authors:** Bin Zhang, Xuchen Cao, Yanxue Liu, Wenfeng Cao, Fei Zhang, Shiwu Zhang, Hongtao Li, Liansheng Ning, Li Fu, Yun Niu, Ruifang Niu, Baocun Sun, Xishan Hao

**Affiliations:** 1 National Key Laboratory of Breast Cancer Prevention and Treatment, Tianjin Medical University Cancer Institute and Hospital (TJMUCIH), Tianjin, P.R.China; 2 Department of Breast Cancer Surgery, Tianjin Medical University Cancer Institute and Hospital (TJMUCIH), Tianjin, P.R.China; 3 Department of Pathology, Tianjin Medical University Cancer Institute and Hospital (TJMUCIH), Tianjin, P.R.China; 4 Department of Breast Cancer Pathology, Tianjin Medical University Cancer Institute and Hospital (TJMUCIH), Tianjin, P.R.China

## Abstract

**Background:**

Experimental evidence suggests that matrix metalloproteinase-13 (MMP-13) protein may promote breast tumor progression. However, its relevance to the progression of human breast cancer is yet to be established. Furthermore, it is not clear whether MMP-13 can be used as an independent breast cancer biomarker. This study was conducted to assess the expression profile of MMP-13 protein in invasive breast carcinomas to determine its diagnostic and prognostic significance, as well as its correlation with other biomarkers including estrogen receptor (ER), progesterone receptor (PR), Her-2/neu, MMP-2, MMP-9, tissue inhibitor of MMP-1 and -2 (TIMP-1 and TIMP-2).

**Methods:**

Immunohistochemistry (IHC) was performed on paraffin-embedded tissue microarray containing specimens from 263 breast carcinomas. The intensity and the extent of IHC were scored by pathologists in blind fashion. The correlation of the gene expression profiles with patients' clinicopathological features and clinical outcomes were analyzed for statistical significance.

**Results:**

MMP-13 protein was detected in the cytoplasm of the malignant cells and the peritumoral stromal cells. MMP-13 expression by tumor cells (*p *< 0.001) and stromal fibroblasts (*p *<0.001) both correlated with carcinoma infiltration of lymph nodes. MMP-13 also correlated with the expression of Her-2/neu (*p *= 0.015) and TIMP-1 (*p *< 0.010), respectively in tumor cells. Tumor-derived, but not stromal fibroblast-derived, MMP-13 correlated with aggressive tumor phenotypes. Moreover, high levels of MMP-13 expression were associated with decreased overall survival. In parallel, the prognostic value of MMP-13 expressed by peritumoral fibroblasts seems less significant. Our data suggest that lymph node status, tumor size, Her-2/neu expression, TIMP-1 and MMP-13 expression in cancer cells are independent prognostic factors.

**Conclusion:**

Tumor-derived, but not stromal fibroblast-derived, MMP-13 correlated with aggressive tumor phenotypes, and inversely correlated with the overall survival of breast cancer patients. MMP-13 may serve as an independent prognostic factor for invasive breast cancer patients. MMP-13 may be particularly useful as a prognostic marker when evaluated along with Her-2/neu and lymph node status.

## Background

Breast cancer is one of the leading causes of cancer death among women worldwide. Of particular significance, the incidence and mortality rate of breast cancer increased sharply in China over the last couple of decades [1]. Clinical parameters, such as the size of the primary tumor, the histological grade, and regional lymph node involvement, are generally useful for predicting the prognosis. However, the profile of molecular markers may provide valuable insights into the underlying mechanisms of disease progression, thus aiding the intervention strategies. In light of the racial disparities of breast cancer incidences and prognoses documented in the literature, it is important to identify and validate specific biomarkers for Chinese breast cancer patients. To this end, estrogen receptor (ER)/progesterone receptor (PR), and oncogene Her-2/neu have been shown to be useful markers of breast cancer [2,3].

Breast cancer mortality derives overwhelmingly from invasion and metastasis, a process that requires continuous and extensive remodeling of tumor stroma. Epithelial basement membrane and extracellular matrix (ECM) are composed of tough network of fibrillar ECM proteins that can be directly degraded only by divalent cation-dependent matrix metalloproteinses (MMPs). Not surprisingly, experimental evidence supports a critical role of MMPs, such as MMP-2 and MMP-9, in the invasion and metastasis of breast cancer [4]. MMP-13, also named collagenase-3, is another MMP that is implicated in the degradation of ECM [5,6]. Recent studies further suggests that MMP-13 may play a central role in the extracellular MMP activation cascade [7,8]. It has been reported that overexpression of MMP-13 in several types of malignancy [9-17] is associated with shorter overall survival of the patients [16,18,19]. Consistently, functional evidence demonstrates that MMP-13 increases the invasive capacities of the malignant cells [20-24]. Interestingly, the clinical utility of MMP-13 as a breast cancer marker remains controversy. While several studies conclude that MMP-13 is produced by tumor stromal fibroblast-like cells [5], others claim that MMP-13 is synthesized predominantly by tumor cells [25]. For example, Balduyck, et al, demonstrated that MMP-13 was expressed in more invasive breast carcinoma cells [21]. Nielsen and colleagues showed that MMP-13 was expressed in myofibroblasts [25,26].

The goal of current study was to evaluate the prognostic values of MMP-13 expression level and its tissue distribution pattern in a large cohort of human breast cancer patients. Our evidence demonstrates that increased tumor-derived MMP-13 expression independently predicts poor prognoses. Furthermore, the combination of MMP-3 with other clinicopathological parameters and/or other breast cancer biomarkers may be particularly useful.

## Methods

### Human sbjects and tssue secimens

The cohort includes a total of 263 cases of invasive breast cancer, diagnosed and surgically treated between January and December 1993 at the Department of Breast Cancer, Tianjin Medical University Cancer Institute and Hospital [27]. Patients had not received prior radiotherapy or neoadjuvant therapies. All patients received conventional postoperative treatments, depending on the extents of the disease. Patients without axillary lymph node involvement were treated with operation alone, while patients with axillary lymph node involvement received six courses of adjuvant chemotherapy with cyclophosphamide/methotrexate/fluorouracil regiment. Patients with positive nodes or tumor size ≥ 5 cm received postoperative radiation. The patients with ER^+^/PR^+ ^tumor were treated for 2–5 years with tamoxifen.

The patient characteristics including age (median: 50.3 years), menopausal status, clinical stage (TNM classification defined by the International Union against Cancer, UICC, 2003) were assessed by the surgical pathologists. These data as well as the postoperative follow-up information including local (regional) recurrence and overall survival have been previously described [27]. Briefly, among the 263 cases, 132 women were premenopausal, while 131 were postmenopausal. At the time of operation, 68 cases (25.9%) were Grade I tumors, 119 (45.25%) cases were grade II tumors, and 76 cases (28.90%) were grade III tumors. We used the commonly used grading standard to assign the scores of histological grades of breast cancer. Briefly, the tumor grading combines nuclear grade, tubule formation and mitotic rate. Grade I is assigned to well-differentiated tumors. Grade II is assigned to moderately differentiated tumors. Grade III is assigned to poorly differentiated tumors. Among the 263 cases, 45 cases (17.1%) were classified of T1, 154 cases (58.6%) were T2, 59 cases (22.4%) were T3 and 5 (1.9%) cases were T4. A total of 128 cases (48.%) were lymph node negative, 57 cases (21.7%) were N1, 26 cases (9.9%) were N2, and 52 case (19.8%) were N3. Local recurrence happened in 8 cases (3%). Metastases were identified in 54 cases (20.5%), including 13 cases to the liver, 12 cases to the bone, 11 cases to the lung, 7 cases to ipsilateral or the contralateral suprascapular lymph nodes, 5 cases to the contralateral breast, 2 cases to the brain, 2 cases to the ovary, 1 case to the pericardium, and 1 case to the pleura. All patients, unless deceased, were followed up for at least 36 months, up to 173 months. The cases lost (due to patients' death or other reasons) for follow-up had been marked as censored data. Patient outcome was defined by the months of post surgery overall survival (OS). This study was performed under a protocol approved by the Tianjin Medical University Administrative Panel on Human Subjects in Medical Research and the Institutional Review Boards of Tianjin Cancer Research institution.

### Tissue mcroarrays (TMA) and imunohistochemistry (IHC)

TMA were constructed with a Beecher Instru-ments Tissue Array (Beecher Instruments, Silver Spring, MD) as previously described [27]. Briefly, archival paraffin blocks of invasive breast cancer cases. The recipient blocks were cut into tissue cores of 0.6 mm in diameter. Two cores of each specimen, representing the tumor areas, are transferred to the donor blocks.

Consecutive 4 μm-thick sections were cut from the recipient blocks to a poly-L-lysine-coated slide for IHC analysis. IHC was performed using a method as described previously [27]. A modification of antigen retrieval (5 min high-power microwave followed by 10 min low-power microwave in phosphate-buffered saline (PBS, PH 7.0) was made for detecting MMP-2, MMP-9, MMP-13, TIMP-1 and TIMP-2. The antibodies and the dilution factors were as follows: MMP-13 (1: 150 diluted Clone VIIIA2 from Lab Vision Corp.), ER (1:450 diluted clone ID5 from DAKO), PR (1:200 diluted clone IA6 from DAKO); Her2/neu (1:1000 diluted polyconal from DAKO), MMP-2 (1:150 diluted polyclonal from Lab Vision Corp), MMP-9 (1:150 diluted polyclonal from Lab Vision Corp), TIMP-1 (1:150 diluted polyclonal from Lab Vision Corp), and TIMP-2 (1:150 diluted polyclonal from Lab Vision Corp).

Human placenta tissue known to express high levels of MMP-2, MMP-9 TIMP-1 and TIMP-2 [28,29] were used as positive controls for these proteins, respectively. Breast cancer cell lines MCF-7 and T47D were used as positive control for ER and PR, respectively. Ovarian cancer cell line SK-OV-3 was used as a positive control for Her-2/neu staining [30]. PBS was used in the place of the primary antibodies in all negative controls of IHC.

### Semi-quantitative masurement of imunostaining

IHC was scored independently using a semi-quantitative scoring system as described below by two pathologists (WC and YL), who were both blinded to patients' clinicopathologic parameters and outcomes. Discordant scores were re-evaluated by the investigators and the consensus scores were used for further analyses. Both the intensity and extent of IHC were assessed [27]. The intensity of the immunostaining was defined by the negative and positive controls as four categories. No brown particle staining: 0; light brown particle in cytoplasm: 1; moderate brown particle: 2; and 3: dark brown particle. The percentage of positive cells, as the extent of immunostaining, was quantified under microscope and classified into four groups. 1: <25% positive cells; 2: 25% to 50% positive cells; 3, 51% to 75% positive cells and 4, >75% positive cells. The staining index (SI), the product of the intensity and the percentage of positive staining, was used to define high (SI ≥ 6) or low (SI < 6) expression of MMP-13, MMP-2, MMP-9, TIMP-1 and TIMP-2.

The criterion of Herceptest/Pathway system was followed to score Her-2/neu Briefly, cases with strong complete membranous staining in more than 10% of the tumor cells were considered strongly positive (+3). Cases with weak to moderate complete membranous staining in more than 10% of the tumor cells were considered moderately positive (+2) and were subsequently confirmed by fluorescence *in situ *hybridization. Cases with little or no membranous staining were considered negative (0 or +1) [31]. The criteria for scoring of ER and PR are similar. Cases with strong/moderate complete nuclear staining in more than 15% of the tumor cells were considered positive, whereas cases with little or no nuclear staining were considered negative [27,31].

### Statistical analysis

Statistical analyses were performed using the SPSS software package 11.0 (SPSS, Inc. Chicago, IL, USA). The correlations between MMP-13 expression and clinicopathologic variables were analyzed using Pearson Chi-square analysis. The same method was used to test the associations of MMP-13 with ER, PR, and Her-2/neu, respectively. Kaplan-Meier curves were constructed for univariate and multivariate analyses using a Cox proportional hazards model to examine the potential prognostic variables on OS. No corrections for multiple comparisons were made. All of the statistical tests were two-sided. *p *values of less than 0.05 were considered statistically significant.

## Results

### Breast cancer cells and peritumoral fibroblasts both express MMP-13

**Figure 1:**
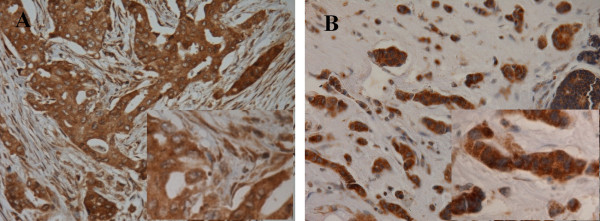
**Differential expression of MMP-13 in breast cancer and peritumoral fibroblast cells**. Invasive ductal carcinoma featured MMP-13 protein in the cytoplasm of both the cancer cells and peritumoral fibroblast cells **(A)**. High levels of MMP-13 protein exclusively in the cytoplasm of the cancer cells **(B)**. The brown color represents the IHC staining of MMP-13. The blue color represents the nuclear counterstain. × 400. Insets of **A **and **B **show a section of the each field with higher magnification. × 1000.

Distinct IHC staining of MMP-13 was obtained from all 263 cases. The tumor or fibroblast-specific staining was semi-quantitatively scored by the SI scales and assigned into high and low categories. As shown in Figure 1, MMP-13 protein expression was detected mainly in cytoplasm of in malignant cells and the peritumoral fibroblasts in 127 (48.29%) and 144 (54.75%) cases, respectively. One hundred and eleven out of 127 specimens with high levels of tumor expression of MMP-13 (87.40%) featured high levels of MMP-13 in peritumoral fibroblasts. Consistently, 106 out of 144 specimens with low levels of tumor expression of MMP-13 were accompanied by peritumoral fibroblasts that expressed MMP-13 at low levels (Table 1). Overall, the detection of cytoplasmic MMP-13 in tumor cells, correlated significantly with the cytoplasmic MMP-3 in the peritumoral fibroblasts (*p *< 0.0001).

**Table 1:** Correlation of tumor-derived MMP-13 with stromal fibroblast-derived MMP-13

		Stromal Fibroblast-derived MMP-13					
		
		Low		High		*p*	*R*
		
		n	%	n	%		
	
Tumor-derived	Low	103	75.7	33	24.3	**< 0.001**	0.634
MMP-13	High	16	12.6	111	87.4		

Low: immunostaining SI < 6; High immunostaining SI > 6; n: number of the cases

### MMP-13 correlates with lymph node infiltration and Her-2/neu expression

To test the potential value of MMP-13 as a breast cancer biomarker, we performed Pearson Chi-square analysis to evaluate the correlation of MMP-13 expression with clinical and histopathological features (tumor size, tumor grade, and lymph node status) that are further stratified as shown in Table 2. *p *values of less than 0.05 was considered statistically significant. High levels of MMP-13 expression, both in cancer cells and in peritumoral fibroblasts, correlated with lymph node metastases (*p *< 0.001), but not with tumor size and histological grade. MMP-13 did not appear to be regulated by sexual steroid hormones, since it was not correlated with the menopausal statuses of the patients. Nor was it correlated with ER or PR. Interestingly, a strong correlation between MMP-13, both in cancer cells (*p *= 0.015) and peritumoral fibroblasts (*p *< 0.001), was observed with Her-2/neu nuclear staining.

**Table 2:** Correlation of high MMP-13 expression with clinicopathological parameters and other biomarkers

		High MMP-13 expression					
		Cancer Cells			Stromal Fibroblasts		
			
Parameters/Markers	Total	*n*	%	*p*	*n*	%	*p*
Menopausal							
Pre-menopausal	132	64	48.5	0.949	70	53.0	0.573
Post-menopausal	131	63	48.1		71	56.5	
Tumor Size							
<2 cm	46	25	54.3	0.365	28	60.9	0.359
>2 cm	217	102	47.0		116	53.5	
Lymph Node Status							
N0	128	43	33.6	**< 0.001**	55	43	**< 0.001**
N1	57	25	43.9		31	54.4	
N2	26	17	65.4		16	61.5	
N3	52	42	80.8		42	80.8	
Histological Grade							
G1	68	22	32.35	**0.001**	27	39.7	0.071
G2	119	56	47.06		67	56.3	
G3	76	49	64.47		51	54.8	
ER Status							
Negative	101	51	50.5	0.572	56	55.4	0.859
Positive	162	76	46.9		88	54.3	
PR Status							
Negative	101	85	52.5	0.086	95	58.6	0.109
Positive	162	42	41.6		49	485	
HER2/neu Protein							
Negative	211	94	44.5	**0.0150**	101	47.9	**< 0.001**
Positive	52	33	63.5		43	82.7	
MMP-2 Protein							
Negative	171	81	47.4	0.234	96	56.1	0.927
Positive	51	29	56.9		29	56.9	
MMP-9 Protein							
Negative	162	88	54.3	0.911	81	50.0	0.367
Positive	58	32	55.2		42	43.3	
TIMP-1 expression							
Negative	131	50	38.2	**< 0.001**	63	48.8	**0.002**
Positive	72	52	72.2		51	70.8	
TIMP-2 Protein							
Negative	41	15	36.6	0.080	52	35.6	**0.029**
Positive	142	74	52.1		34	51.5	

### MMP-13 correlated with TIMPs, but not with MMP-2 and MMP-9

**Figure 2:**
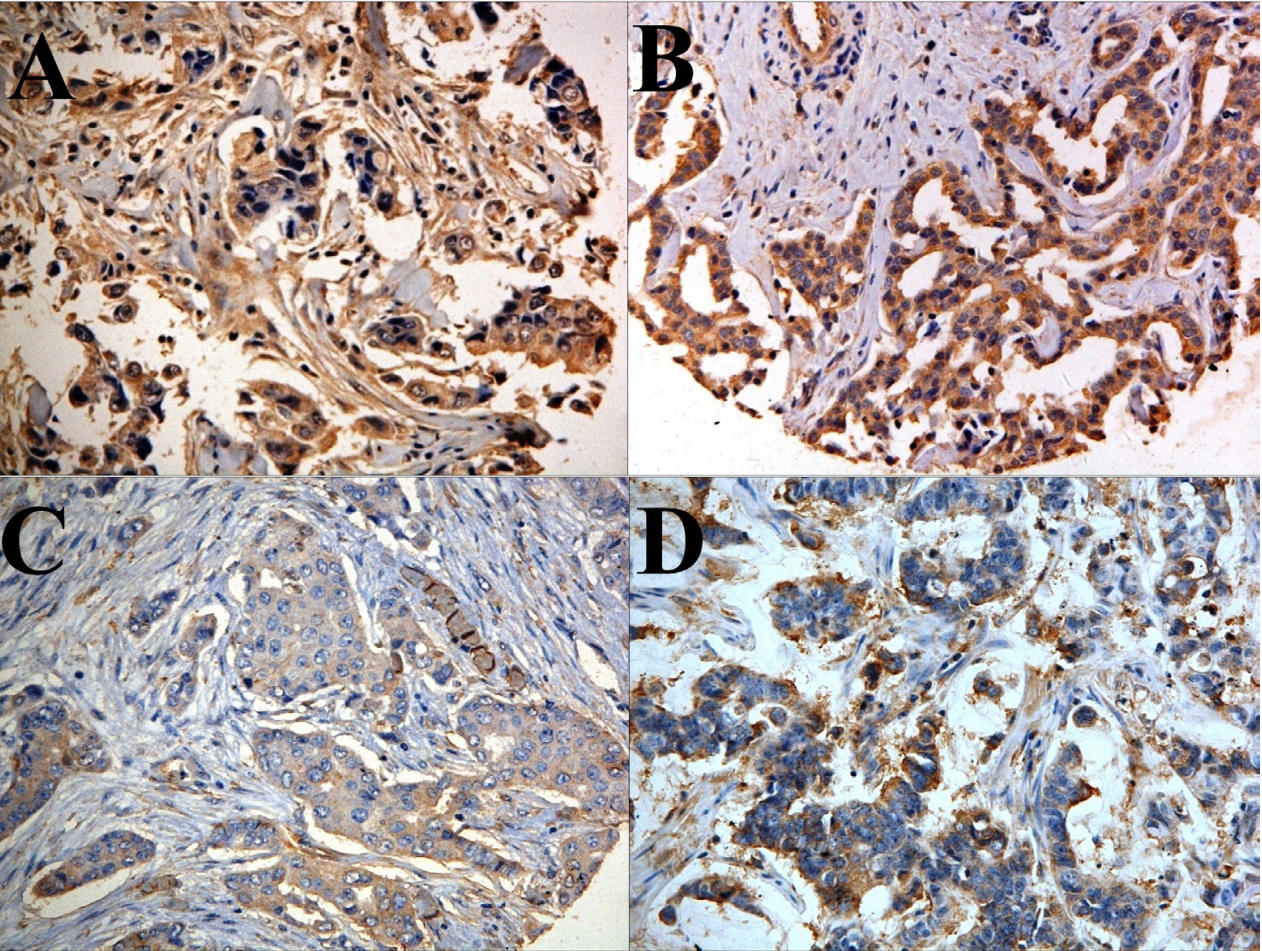
**Representative IHC of an infiltrating ductal carcinoma TMA**. **(A): **MMP-2; **(B): **MMP-9; **(C)**: TIMP-1; and **(D)**: TIMP-2. The brown staining represents the indicated antigen, while blue color is the counterstain of the nuclei. ×400.

Biochemically redundant members of the MMP family may have intricate interplay in tumor progression. To begin to understand the relative significance of MMP-13, we examined its correlation with other MMPs (MMP-2 and MMP-9) and tissue inhibitors of MMP (TIMP-1 and TIMP-2). Distinct IHC staining of each of these proteins were detected in the cytoplasm of both the cancer cells and adjacent fibroblasts (Figure 2). MMP-13 detected either in cancer cells or in adjacent fibroblasts, was not correlated with MMP-2 or MMP-9 (Table 2). Thus, the differential expression of MMP-13 in breast tumor progression may be regulated by a mechanism different from those for MMP-2 and MMP-9. High levels of MMP-13 in both cancer cells (*p *< 0.010) and peritumoral fibroblasts (*p *= 0.002), significantly correlated with the levels of TIMP-1 protein in the same specimens. In addition, MMP-13 in peritumoral fibroblasts correlated to lesser extent, albeit significant, with the levels of TIMP-2 protein (*p *= 0.029).

### MMP-13 negatively correlates with breast cancer OS

**Figure 3:**
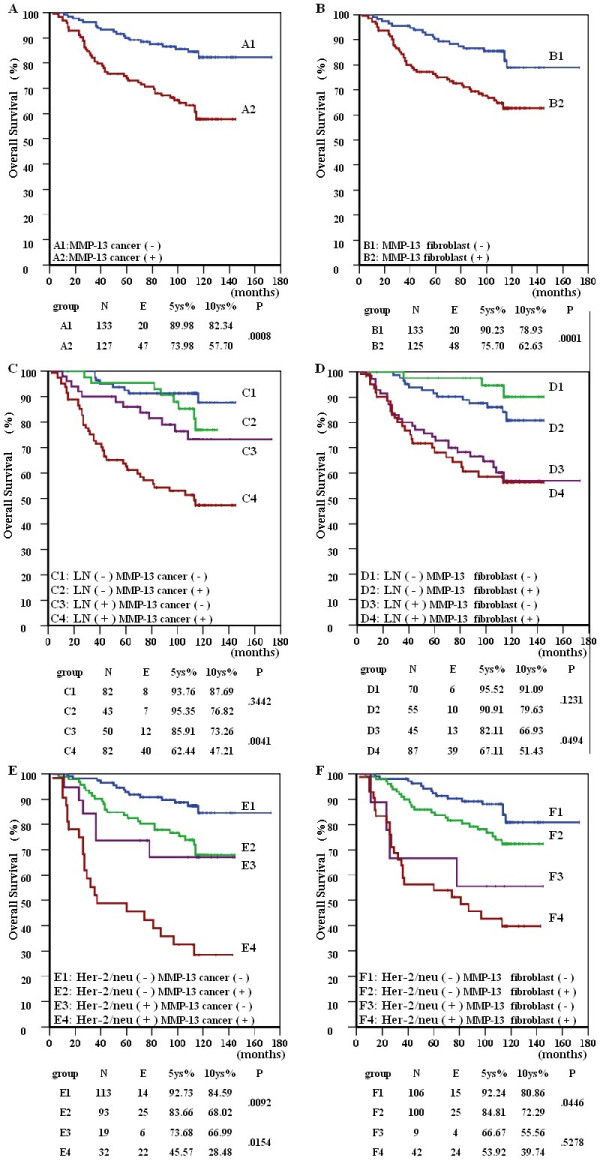
**The prognostic significance of high MMP-13 protein expression in the entire patient population**. The graphs show the effect of MMP-13 expression within cancer cells (**A**) and within peritumoral fibroblast cells (**B**) on patient overall survival (Log-rank test) and stratified analysis by lymph nodes status (**C **&**D**) and Her-2/neu status (**E **&**F**). Both high MMP-13 expression in cancer cells (**A**) and fibroblasts (**B**) associated with decreased overall survival (*p *= 0.0008 and 0.0001, respectively). When patients' OS were stratified by lymph node status and Her-2/neu status, MMP-13 expression in cancer cells was associated with reduced OS in the Her-2/neu positive and negative (**E**, *p *= 0.0092 and *p *= 0.0154, respectively) and lymph node positive subgroups (**D**, *p *= 0.0041). However, MMP-13 expressed within fibroblasts only weakly affected OS of lymph node positive (**D**) and Her-2/neu negative subgroups (**F**, *p *= 0.04694 and 0.04461, respectively)

The mean follow-up of the 263 patients in our cohort is 92.1 months. Kaplan-Meier analyses showed a clear stratification of the OS between the patients with high levels of MMP-13 expression and the patients with low levels of MMP-13 expression (Table 3). High levels of MMP-13 expression both in the cancer cells (*p *= 0.0008) and the neighboring fibroblasts (*p *= 0.0001) negatively correlated with OS (Figure 3A and 3B). Tumor expression of MMP-13 negatively correlated with patients' OS regardless of the Her-2/neu status. It was noted that MMP-13 detected in peritumoral fibroblasts correlated with the OS of the lymph node positive subgroup (*p *= 0.047) and with the Her-2/neu negative subgroups (*p *= 0.045) (Figure 3C–F). When the patients' survival was stratified by lymph node status, MMP-13 expression was negatively correlated with the OS of the lymph node positive subgroups (*p *= 0.0041), but not with the OS of the lymph node negative subgroups (*p *= 0.344).

**Table 3:** Correlation of MMP-13 expression with OS of overall and stratified subpopulations.

	MMP-13SI	Tumor				Stromal Fibroblast			
		
		n	E	OS (95% CI)	*p*	n	E	OS (95% CI)	*p*
**Overall**									
	Low	133	20	153 (145~161)	**0.0008**	132	20	153(145~161)	**0.0001**
	High	127	47	107 (98~116)		125	48	106 (97~115)	
**Node Status**									
Negative	Low	82	8	135 (128~142)	0.3442	70	6	137 (131~143)	0.1231
	High	43	7	122 (115~129)		55	10	128/(119~137)	
Positive	Low	50	12	142 (127~158)	**0.0041 **	45	13	136 (118~153)	**0.0494**
	High	82	40	93 (80~105)		87	39	97 (85~108)	
**HER2/neu**									
Negative	Low	113	14	157 (149~165)	**0.0092**	106	15	155 (145~163)	**0.0446**
	High	93	25	120 (111~129)		100	25	121 (113~130)	
Positive	Low	19	6	109 (85~133)	**0.0154**	9	4	96 (58~133)	0.5278
	High	32	22	69 (50~88)		42	24	81 (64~98)	

OS: Months of overall survival; n: Number of patients; E: Events of positive stains; CI: confidence interval.

In the Cox regression model, univariate survival analyses showed that tumor size, lymph node positivity (>N2), PR, Her-2/neu, TIMP-1 and MMP-13 were each associated with worse prognoses, while, multivariate survival analyses showed that lymph node positivity (>N2), tumor size, Her-2/neu, MMP-13 and TIMP-1 expression by cancer cells were independent adverse prognostic factors for overall survival (Table 4). Parallel analyses showed that ER and Tumor Grades were not correlated with OS.

**Table 4:** Univariate and multivariate Cox survival analysis

Variables	*p*	HR	OS (95% CI)
**Univariate **			
Menopausal Status	0.215	0.958	(0.772–1.188)
Tumor Size	**0.029**	1.569	(1.264–1.946)
Lymph Node Status	**< 0.001**	2.198	(1.860–2.596)
Histological Grade	**0.045**	1.438	(1.072–1.929)
ER	0.091	0.778	(0.579–1.042)
PR	**0.046**	0.687	(0.565–0.836)
Her-2/neu	**< 0.001**	1.989	(1.684–2.349)
High Tumor MMP-13	**0.007**	1.357	(1.171–1.571)
High Stromal Fibroblast MMP-13	0.146	0.995	(0.786–1.259)
High Tumor-derived MMP-2	0.443	1.276	(0.969–1.678)
High Tumor-derived MMP-9	0.889	1.245	(0.984–1.575)
High Tumor-derived TIMP-1	**0.023**	1.278	(1.103–1.480)
High Tumor-derived TIMP-2	0.881	1.072	(0.842–1.364)

**Multivariate**			
Histological Grade	**0.041**	1.441	(1.074–1.933)
Lymph Node Status	**< 0.001**	2.199	(1.861–2.598)
Her-2/neu	**< 0.001 **	1.98	(1.685–2.350)
Tumor Size	**0.024 **	1.570	(1.266–1.948)
High Tumor MMP-13	**0.006 **	1.565	(1.178–1.581)
High Tumor TIMP-1	**0.021**	1.281	(1.106–1.484)

HR: Hazard Risk in Cox proportional hazard model analysis. HR>1.0 means positive correlation with risk. HR<1.0 means negative correlation with risk.

## Discussion

Predicting patients' prognoses is clearly one the most challenging issues in breast cancer treatments. Racial, geographical and dietary factors have all been considered for their impacts on breast cancer incidences and survival. However, at the histopathological level, breast cancer is a highly heterogeneous disease, hampering the prognosis predictions. Histological grading, as shown by others and data in this paper, is not a reliable prognostic predictors. To this end, hormone receptors (ER and PR) and Her-2/neu have been used with certain degrees of success as the biomarkers. In the current study, we examined a cohort of 263 Chinese breast cancer specimens and report the evidence that MMP-13 correlated with more aggressive breast cancer. Our data suggest that MMP13 may serve as a marker for poor prognoses. To our knowledge, this is the first IHC study to investigate the potential utility of MMP-13 as a biomarker of breast cancer among Chinese patients.

Our data support the earlier notion that MM-13 marked the transition of ductal carcinoma *in situ *to invasive carcinomas [25] and increased the invasiveness of breast cancer cells *in vitro *[21]. The biological activity of MMP-13 in tumor progression may not be limited to breast cancer. For instance, Culhaci N et al, showed a significant correlation of MMP-13 with the aggressiveness of head and neck squamous carcinomas [20].

Although tumor cells are the ones that pass through multiple tissue barriers to metastasize, increasing evidence demonstrates a critical role of stromal cells in tumor microenvironments [32,33]. Vizoso FJ noted that MMP-13 in fibroblastic cells and mononuclear immune cells was associated with increased risks of metastases [34]. Interestingly, others have reported that MMP-13 protein was restricted to small stromal foci within the tumor masses and was specifically produced by invading tumor cells [25]. In this study, we found MMP-13 protein in the cytoplasm of both cancer cells and tumor-adjacent fibroblasts. The expression of MMP-13 in cancer cells correlated with the MMP-13 expression in the peritumoral fibroblasts. Furthermore, high levels of MMP-13 in cancer/peritumoral fibroblast cells correlated with tumor infiltration of lymph nodes. While this finding suggests that MMP-13 is likely to play a role in promoting tumor invasion and metastasis, future studies are needed to clarify whether the MMP-13 protein was expressed by both cancer cells and fibroblasts or by tumor cells alone.

The two types of most widely accepted prognostic biomarkers for breast cancer are hormone receptor (ER and/or PR) and oncoprotein Her-2/neu. ER is used to predict not only the response to endocrine therapy but also better overall survival. In contrast, Her-2/neu predicts more aggressive tumor phenotype, poor disease-free survival and poor OS [26,27]. High levels of MMP-13 in cancer cells correlated with the expression of the Her-2/neu protein. Recently, similar results were reported by Ocharoenrat et al, with head and neck squamous cell carcinoma specimens [35]. Currently, it is not known whether MMP-13 is regulated by Her-2/neu. To this end, it is important to note that MMP-13 seems to be of prognostic value even for the Her-2/neu positive subset of cases, suggesting a Her-2/neu-indepdent function of MMP-13 in promoting breast cancer progression.

The correlation of MMP-13 with poor OS and tumor infiltration of lymph nodes suggests that it may promote tumor invasion. Since MMP-13 is a metalloproteinase, it may act in a similar manner as other MMPs, such as MMP-2, MMP-9. In fact, both MMP-2 and MMP-9 have been extensively studied as biomakers and as well as therapeutic targets in breast cancer [36-39]. Interestingly, MMP-13 in both cancer cells and peritumoral fibroblast cells showed no correlation with either MMP-2 or MMP-9. Instead, we noted that MMP-13, both in cancer cells and peritumoral fibroblast cells, correlated with TIMP-1 and TIMP-2 (to a lesser extent). Thus, MMP-13 may be independent of MMP-2 and MMP-9 as a marker as well as a regulator of breast tumor progression. The intricate interplays among MMPs and TIMPs could be more complex. Indeed, in contrast to the initial simple biochemistry-based hypothesis, TIMP-1 is viewed as a promising marker to predict poor prognoses of human breast cancer [40-42]. The recent discovery of proteolysis-independent biological functions of TIMPs, such as the growth-stimulating, anti-apoptotic and pro-angiogenic properties, provided new insights into this paradox [40-42]. Further studies are needed to clarify whether MMP-13 and TIMPs act as cognate partners or independent of each other.

## Conclusion

This IHC study of TMA with 263 specimens of Chinese breast cancer provided the first evidence that MMP-13 is highly expressed by both the tumor cells and adjacent fibroblast cells. High levels of MMP-13 in these two tissue compartments were strongly correlated with each other, and further correlated with Her-2/neu, TIMP-1, lymph node metastasis and decreased overall survival. Our study suggests a potential application of MMP-13 as an independent biomarker for breast cancer prognosis.

## Abbreviations

MMP: matrix metalloproteinase; TIMP: tissue inhibitor of matrix metalloproteinase; ER: Estrogen Receptors; PR: Progesterone Receptors; ECM: extracellular matrix; RFS: relapse-free survival; OS: Overall survival; SI: staining index

## Competing interests

The author(s) declare that they have no competing interests.

## Authors' contributions

ZB, CXC and ZF participated in the design of the study, optimized and carried out the immunohistochemical staining, performed the statistical analysis and drafted the manuscript. LHT and NLS collected the human tissue. LYX and CWF evaluated the results of immunohistochemical staining. ZSW and SBC prepared tumor tissue arrays block. FL and NY classified the invasive carcinomas. HXS and NRF participated in the design and the coordination of the study. All authors read and approved the final manuscript.

## Pre-publication history

The pre-publication history for this paper can be accessed here:


